# Phytochemical Screening, Toxic Effects, and Antimicrobial Activity Studies of *Digitaria abyssinica* (Hochst. ex A.Rich.) Stapf (Poaceae) Rhizome Extracts against Selected Uropathogenic Microorganisms

**DOI:** 10.1155/2023/4552095

**Published:** 2023-01-05

**Authors:** William Lemayian Sapunyo, James Mucunu Mbaria, Laetitia Wakonyu Kanja, Meshack Juma Omolo, Jared Misonge Onyancha

**Affiliations:** ^1^Department of Public Health, Pharmacology and Toxicology, College of Veterinary and Agricultural Sciences, University of Nairobi, P.O. Box 29053-00625, Nairobi, Kenya; ^2^Kenya Aids Vaccine Initiative Institute of Clinical Research-(KAVI-ICR), P.O. Box 19676-00202, Nairobi, Kenya; ^3^Department of Pharmacognosy, College of Health Sciences, Mount Kenya University, P.O. Box 342-01000, Thika, Kenya

## Abstract

In Kenya, the *D. abyssinica* rhizome's decoction is traditionally used to treat urinary tract infections (UTIs), mainly gonorrhea and candidiasis. UTIs are the most severe public health problems that affect over one hundred and fifty million people worldwide annually. They are caused by a wide range of microorganisms where *Escherichia coli* is known to be the main causative pathogen. Medicinal plants are used in traditional Kenya set up for treatment and most recently as an alternative source of treatment for UTIs due to the increased cost of treatment and many challenges experienced with antibiotic therapy. The current study is designed to investigate the phytochemical composition, acute oral toxicity, and antimicrobial activity of *Digitaria abyssinica* rhizome extracts against *Staphylococcus aureus*, *Escherichia coli*, *Neisseria gonorrhea*, and *Candida albicans*. The rhizomes of *D. abyssinica* were obtained, dried, ground, and extracted using water and organic solvents. The phytochemical assay was carried out using standard phytochemical screening methods. Single-dose toxicity studies were done to determine LD_50_ while disk diffusion and microbroth dilution techniques were used to determine antimicrobial activity. Results revealed that saponins, phenolics, alkaloids, cardiac glycosides, tannins, flavonoids, steroids, and terpenes were present in the powder, aqueous, methanol, and dichloromethane : methanol extracts. All the extracts had an LD_50_ of above 2,000 mg/kg of body weight and there was no observation of behavioral changes. Also, the aqueous and methanol extracts revealed antifungal activity against *Candida albicans* with the lowest average minimum zone of inhibition at MIC of 31.25 mg/ml. The study did not reveal antibacterial activity for any extract against the studied uropathogenic bacteria, *Staphylococcus aureus*, *Escherichia coli*, and *Neisseria gonorrhoeae.* The results from the current study suggested that *D. abyssinica* rhizome aqueous and methanol extracts have potential antifungal activity against *C. albicans*, thus validating the folklore of its use to treat candidiasis.

## 1. Introduction

Urinary tract infections (UTIs) are termed severe public health problems and affect over one hundred and fifty million people worldwide per year [1]. UTIs are known to affect more than half of women at least once in their lives and reinfection is reported to be more recurrent in young women [2]. Urinogenital infections are caused by bacteria and fungi, the most causative agent is *Escherichia coli* usually called uropathogenic *Escherichia coli* (UPEC) [3]. Other pathogenic microorganisms are *Klebsiella pneumoniae*, *Proteus mirabilis*, *Enterococcus faecalis*, group B *Streptococcus* (GBS), *Staphylococcus saprophyticus*, *Staphylococcus aureus*, and *Candida* species [4]. The most recommended antibiotics for the treatment of UTIs are trimethoprim, sulfamethoxazole, ciprofloxacin, and ampicillin. Nevertheless, increasing rates of antibiotic resistance and high recurrent rates and the spread of multidrug resistance (MDR) threaten to greatly enhance the burden that these common infections place on society [5]. There is a continuous effort toward the development of alternative therapies that can be used to manage drug resistance [3, 6]. A large population of over 80% in low-income countries uses medicinal plants as an alternative source of antibiotics and other conditions for primary health care [7–9]. The World Health Organization advocates the use of medicinal and aromatic plants as an alternative source of drugs that can be potentially effective in the treatment of uropathogens [10, 11]. Some plants have demonstrated antimicrobial activities [8, 10–14].

Scientific literature has been published about the plant extracts of the Poaceae family that have chemicals with antimicrobial activity [15–18]. Some of the Poaceae plants that have demonstrated *in vitro* antibacterial activities are *Cynodon dactylon*, *Cymbopogon citratus*, *Triticum aestivum*, *Bambusa vulgaris*, *Dichanthium annulatum*, *Dactyloctenium aegyptium*, *Imperata cylindrica*, *Eleusine indica*, *Saccharum spontaneum*, and *Vetiveria zizanioides* [19–21]. In addition, *Panicum* maximum and *Cymbopogon citratus* have that they comprise secondary metabolites that are active against fungal strains [22, 23]. The activity of Zea mays silk extracts is also known for the treatment of uropathogenic microorganisms [24]. The current study focuses on *D. abyssinica* (Poaceae) referred to as East African couch grass [25]. It is a common weed that is used as food for livestock characterized by low nutritional value [26, 27]. Different parts of the plant are used traditionally to treat flu and diarrhea [28], liver diseases [29], hernia [30], malaria, yellow fever, and wound healing [28, 31]. In Kenya, the *D. abyssinica* rhizome's decoction has been traditionally used to treat urinary tract infections, mainly gonorrhea and candidiasis [32]. There is limited scientific evidence on the biological activities of *D. abyssinica*. Based on the ethnomedicinal claims of *D. abyssinica*, the current study seeks to evaluate the safety, phytochemistry, and antimicrobial activity of *D. abyssinica* rhizome extracts against the selected uropathogenic microorganisms, *Staphylococcus aureus*, *Escherichia coli*, *Neisseria gonorrhea*, and *Candida albicans*. The study aims to validate the ethnomedicinal claims of using the extracts of *D.* abyssinica rhizome by traditional medicine practitioners and herbalists.

## 2. Materials and Methods

### 2.1. Chemicals, Reagents, and Drugs

The chemicals for extraction and preparation of extractions, absolute ethanol (AR), absolute methanol (AR), and dimethyl sulfoxide (DMSO) were from Loba Chemie Pvt., India. Mueller Hinton agar, Mueller Hinton broth, and GC agar media were supplied by HiMedia Laboratories Pvt. Ltd, India. While VCNT and vitox were from Oxoid Limited. Antibacterial sensitivity disks ciprofloxacin, cephalexin, azithromycin, tetracycline, and ceftriaxone sensitivity disks (Liofilchem) and antifungal sensitivity disks: clotrimazole, econazole, ketoconazole, miconazole, and nystatin (Sanofi Diagnostics Pasteur) were used in this study. The chemicals and reagents were of analytical grade.

### 2.2. Collection of Plant Materials

The fresh rhizomes of *D. abyssinica* were collected from Mua, Kyaani, in Machakos county in January 2021 with the help of Mr. Musembi Kimeu, a plant taxonomist at the University of Nairobi, Land Resource Management and Agricultural Technology (LARMAT). The plant materials were transported to the Department of Public Health, Pharmacology, and Toxicology (PHPT), University of Nairobi and were identified and authenticated by Ms. Carol Kyalo of the University of Nairobi, Land Resource Management and Agricultural Technology (LARMAT). A voucher specimen number LARMAT/Herb/Da was assigned and deposited in the LARMAT herbarium for ease of reference.

### 2.3. Extraction of the Plant Material

The fresh rhizome of *D. abyssinica* was cleaned with clean water, then air-dried in a well-ventilated, insect- and rodent-free at room temperature. The rhizomes were pulverized into powder by use of an electric mill. The resulting powder was kept in a well-labeled manilla sack and kept in a cool and nonhumid place awaiting extraction.

Aqueous extraction was prepared by cold maceration by soaking 300 g of ground powder in 2.5 liters of distilled water. The extraction mixture was stirred continuously and allowed to macerate for 48 hours and then filtered through a Whatman No. 1 filter paper. The resultant filtrate was then lyophilized using a freeze dryer. The obtained freeze-dried product was weighed and stored in airtight plastic vials at 4°C in a refrigerator awaiting further analysis.

The methanolic extract was prepared by measuring 200 g of *D. abyssinica* rhizome extract into an extraction jar, adding 1 liter of analytical methanol gradually and then shaking vigorously for 48 hours to macerate. The process was repeated for another batch of 200 g of rhizome powder. The resultant mixture was then decanted and filtered through cotton gauze to remove course residues. The resultant filtrate was then filtered through a Whatman No. 1 filter paper. The resultant filtrate was then combined and reduced in a vacuum at 50°C using a rotary evaporator. To further remove the solvent and concentrate the extract, the extract was placed in a clean, dry, and light-resistant bottle and placed in a sand bath set at 35°C. Finally, it was weighed using an analytical balance, and the percentage yield of the extract was calculated and stored at 4°C in a refrigerator awaiting further analysis.

The dichloromethane : methanol (1 : 1) extract was prepared by measuring 200 g of rhizome powder into an extraction jar. An equal amount of dichloromethane and methanol was mixed to make a solution of 1 liter. The mixture was then gradually added to the powder. The mixture was shaken vigorously for 48 hours and allowed to macerate. The abovementioned process was then repeated for another batch of 200 g of the rhizome powder. The resultant mixture was then decanted and then filtered through gauze. The resultant mixture was then filtered through a Whatman No. 1 filter paper. The resultant mixture from the first and the second batch was combined and reduced in a vacuum (in vacuo) at 50°C using a rotary evaporator. To further remove the solvent and concentrate the extract, the extract was placed in a clean, dry, and light-resistant bottle and placed in a sand bath set at 35°C. Finally, it was weighed using an analytical balance and stored at 4°C in a refrigerator until use.

### 2.4. Experimental Animals

Fifteen 8–10-week, female *Wistar* albino rats weighing 125 ± 45 grams were used to assess the acute oral toxicity of *Digitaria abyssinica* rhizome extracts. These animals were purchased from the Kabete Vet Lab animal house. They were transported to the Public Health, Pharmacology, and Toxicology (PHPT) Department animal house where they were housed for 5 days to acclimatize following Biosafety, Animal Use and Ethical Committee (BAUEC) guidelines. All the experimental animals were nulliparous and nonpregnant. They were housed at a temperature of 25 ± 3°C and 56–60% relative humidity. A 12-hour day and night cycle were maintained, and the animals were fed on standard rat pellets from a commercial feed supplier (Unga Feeds). Water was provided ad libitum.

### 2.5. Phytochemical Screening

The analysis of the phytochemical groups of compounds, namely, plant flavonoids, tannins, saponins, phenols, coumarins, steroids, terpenoids, glycosides and alkaloids of water, methanol, and dichloromethane : methanol (1 : 1) extracts of *D. abyssinica* rhizomes were done using standard phytochemical screening procedures described by [33, 34] as modified by [35]. The tests were performed in triplicates to ensure the results' accuracy and were examined by visual observations.

#### 2.5.1. Test for Saponins (Froth Test)

About 0.1 g of the water, methanol, and dichloromethane : methanol (1 : 1) extracts of *D. abyssinica* rhizome were added to 10 ml of distilled water in separate test tubes, respectively. The mixtures were boiled for 10 minutes, and they were filtered using Whatman filter paper No.1. A mixture of 3 ml distilled water and 5 ml of the filtrate was agitated vigorously for 15 seconds and left to stand for 10 minutes. Frothing which persisted for about 3 minutes was an indication of saponins [36].

#### 2.5.2. Test for Alkaloids

Two tests, namely, Mayer's and Dragendorff's tests, were done to detect alkaloids in the extracts.

(1) *Mayer*'*s Test*. Approximately, 0.1 g of water, methanol, and dichloromethane : methanol (1 : 1) extracts of *D. abyssinica* rhizome were mixed with 5 ml of 1% HCl in separate test tubes, respectively; each mixture was warmed and then filtered through Whatman filter paper No.1. Two drops of Mayer's reagent (mercuric potassium iodide) were added to 2 ml of water, methanol, and dichloromethane : methanol (1 : 1) extracts. The appearance of a cream-colored precipitate indicates the presence of alkaloids [37, 38].

(2) *Dragendorff*'*s Test*. The test was carried out by adding two drops of Dragendorff's reagent (potassium bismuth iodide solution) to 2 ml of the filtered water, methanol, and dichloromethane : methanol (1 : 1) extracts in separate test tubes. A reddish-brown precipitate indicates the presence of alkaloids [37, 38].

#### 2.5.3. Test for Flavonoids (Sodium Hydroxide Reagent Test)

Approximately, 0.1 g of the water, methanol, and dichloromethane : methanol (1 : 1) extracts of *D. abyssinica* rhizome were warmed in 10 ml of 70% ethanol and thereafter hydrolyzed with 10% hydrochloric acid. Sodium hydroxide (10%; 1 ml) was added to the mixture and the appearance of yellow color was a positive test for the presence of flavonoids [39, 40].

#### 2.5.4. Test for Phenolics

Approximately, 0.1 g of the water, methanol, and dichloromethane : methanol (1 : 1) extracts of *D. abyssinica* rhizome were measured and put into separate test tubes and 10 ml of 70% ethanol were added. The mixtures were boiled using water for five minutes. The extracts were then cooled, and they were filtered through Whatman filter paper No.1. Five drops of 5% of ferric chloride were added into 2 ml of each respective extract. The formation of a green precipitate indicates the presence of phenols [36].

#### 2.5.5. Test for Glycosides

(1) *Keller*–*Killiani Test*. Glacial acetic acid (4.0 ml) solution with 1 drop of 2.0% FeCl_3_ mixture was added to the 10 ml water, methanol, and dichloromethane : methanol (1 : 1) extracts of *D. abyssinica* rhizome in separate test tubes. One milliliter of concentrated sulphuric acid was added to the mixture and a reddish-brown ring formed between the layers which progressively turned blue indicating the presence of steroidal glycosides with deoxy sugars [36].

(2) *Kedde Test*. One milliliter of 2% solution of 3,5-dinitrobenzoic acid in 95% alcohol was added to the 2 ml of the water, methanol, and dichloromethane : methanol (1 : 1) extracts of *D. abyssinica* rhizome. The solution was made alkaline with 5% sodium hydroxide. The appearance of a purple-blue color indicates the presence of an unsaturated lactone ring in cardenolides [36].

#### 2.5.6. Test for Steroids

(1) *Salkowski*'*s Test*. Approximately, 2 mg of the water, methanol, and dichloromethane : methanol (1 : 1) extracts of *D. abyssinica* rhizome were dissolved in 1 ml of chloroform and then shaken gently. Five drops of concentrated sulphuric acid were added along the side of the test tube. A reddish-brown color that was formed at the interface indicated steroids [39, 41].

(2) *Liebermann*–*Burchard Test*. About 2 mL of acetic acid was added to 1 mL of the water, methanol, and dichloromethane : methanol (1 : 1) extracts. After cooling the solution in an ice bath, concentrated sulphuric was added carefully. The development of violet to blue or bluish-green color confirms the test for steroids [42, 43].

#### 2.5.7. Test for Terpenoids (Salkowski's Test)

Approximately, 2 mg of the water, methanol, and dichloromethane : methanol (1 : 1) extracts of *D. abyssinica* rhizome were dissolved in 2 mL chloroform along with concentrated sulphuric acid. The red-brown color at the interface indicates terpenoids [37, 42].

#### 2.5.8. Test for Coumarins

Approximately, 0.5 g of the extracts and powder of *D. abyssinica* were added into separate test tubes. The test tubes were covered with filter paper which was moistened with 1 N NaOH. The tubes were warmed in a hot water bath and then allowed to cool. Yellow fluorescent color was an indication of coumarins [44].

#### 2.5.9. Test for Tannins

About 0.5 g of the sample was boiled in 20 ml of water and filtered. 0.1% of ferric chloride was added to the filtrate. The formation of a brownish-green or blue-black color was an indication of the presence of tannins [44].

### 2.6. Single-Dose Toxicity Study

The up-and-down procedure for acute oral toxicity described by the Organization for Economic Cooperation and Development (OECD), [45] Document No. 425 was used to determine the safety of the aqueous, methanol, and dichloromethane : methanol (1 : 1) extracts of *D. abyssinica* rhizome. Five female Wistar rats were used to perform the limit tests for each of the aqueous extracts. A dose of 2000 mg/kg body weight of each extract was given orally to one female rat, and fatalities were not observed; thereafter, four additional animals were dosed sequentially. In addition, the rats were observed for wellness parameters that included the skin and fur appearance, fecal matter consistency, urination and urine appearance, itching, salivation, convulsions, tremors, breathing, coma somatomotor activity, aggression, grooming, eyes, and unconsciousness or death. The obsevations were made at time intervals of 30 minutes, 4 hours, 24 hours, 48 hours, 7 days, and 14 days. The procedure was repeated to evaluate the methanol and dichloromethane : methanol (1 : 1) extracts too.

### 2.7. Antimicrobial Studies

#### 2.7.1. Test Microorganisms

A fungal microorganism and 3 bacterial strains were obtained from the stock cultures from the University of Nairobi, Medical Microbiology Department Laboratories. The standard bacterial strains of *Staphylococcus aureus* (ATCC 29213), *Escherichia coli* (ATCC 25922), and clinical isolates of *Neisseria gonorrhea* and *Candida albicans* were used in this study.

#### 2.7.2. Preparation of Inoculums

To prepare the stock cultures, CLSI guidelines and procedures were used. The method as described by [46] was used with modifications. In brief, bacterial stock cultures were subcultured on respective media and incubated at 37°C for 24 hours. To obtain young growing culture, Neisseria gonorrhea isolates were cutured on Thayer Martin agar while isolates of *Escherichia coli* and *Staphylococcus aureus* were cultured on MacConkey and sheep blood agar, respectively.* Candida albicans* were subcultured in Sabouraud Dextrose Agar (SDA) at 37°C for 48 hours. The test strains were suspended in Mueller–Hinton broth (MHB) to give a final density of 1.5 × 10^6^ bacteria colony-forming units and 1.5 × 10^5^ fungal colony-forming units.

#### 2.7.3. Preparation of the Stock Solutions

Six different concentrations of 15.625 mg/ml, 31.25 mg/ml, 62.50 mg/ml, 125 mg/ml, 250 mg/ml, and 500 mg/ml of each of the three extracts (aqueous, methanol, and dichloromethane : methanol (1 : 1) were prepared for susceptibility testing using 1% DMSO as a diluent. A vortex mixer was used to facilitate the dissolving of extracts into the 1% DMSO. A constant volume of 20 *μ*l of each of the individual stock solutions was pipetted using a micro titer-pipette onto sterile filter paper disks measuring 6 mm to prepare the respective concentrations of the plant extracts. 20 *μ*l of 1% DMSO was impregnated onto sterile filter paper disks which were used as the negative control for the experiment.

#### 2.7.4. Disk Diffusion Method

Antimicrobial activity was evaluated using the disk diffusion method as described by [47] with modifications. Appropriate agar plates were inoculated with respective isolates of the test microorganism. Sterile filter paper disks (6 mm in diameter) containing each of the 3 extracts at the desired concentration of (15.625, 31.25, 62.50, 125, 250, and 500) mg/ml were placed on the surface of the agar, using sterile forceps. 1% dimethyl sulfoxide (DMSO) was used as the negative control. Cephalexin (30 *μ*g), ciprofloxacin (5 *μ*g), and tetracycline (30 *μ*g) sensitivity disks were used as a control against *Staphylococcus aureus*; cephalexin (30 *μ*g), ciprofloxacin (5 *μ*g), and tetracycline (30 *μ*g) sensitivity disks were used as a control against *Escherichia Coli*, cephalexin (30 *μ*g), and azithromycin (15 *μ*g) sensitivity disks control susceptibility of *Neisseria gonorrhoeae* while clotrimazole (50 *μ*g), econazole (50 *μ*g), ketoconazole (50 *μ*g), miconazole (50 *μ*g), and nystatin (100 *μ*g) disks were used as control of the extract against *Candida albicans.*

The Mueller–Hinton agar plates inoculated with *S. aureus* and those inoculated with *E. coli* were incubated at 37°C for 24 hours. Thayer Martin Media inoculated with *Neisseria gonorrhoeae* was incubated at 5% CO_2_ for 48 hours while Sabouraud Dextrose Agar inoculated with *Candida albicans* was incubated at 37°C for 24 hours. Generally, the antimicrobial agent diffuses into the agar and inhibits the germination and growth of the test microorganism.

After the incubation period, the diameters of the inhibition zones were measured in millimeters using a transparent ruler. All the tests were done in triplicates and the means were calculated as the results.

#### 2.7.5. Broth Macrodilution Technique

The broth macrodilution procedure as described in 2021 by Mailu et al. [46] with modification was used to determine the minimum inhibitory concentration for the active crude extracts against the test microorganisms. Six culture tubes with 2 ml sterile Mueller–Hilton broth were prepared. From the stock solution, two-fold serial dilutions were prepared. 0.1 ml of each microorganism was inoculated into each tube of diluted plant extract using a micropipette. The bacterial organisms and the fungal organism were then incubated for 24 hours at 37°C. The extract's minimum inhibitory concentration (MIC) value was determined by observing the lowest concentration of plant extracts that prevented the visible growth of microorganisms resulting in no visible growth (turbidity).

To determine MBC, all broth in tubes with no visible bacterial growth was aseptically cultivated in sterile agar using the streak-plate method and incubated at appropriate temperatures and conditions. The MIC value is the lowest concentration of the plant extract that demonstrates no visible bacterial growth. All tubes with no visible fungal growth were aseptically cultured in sterile molten agar and incubated using the streak-plate method to determine the minimum bactericidal concentration (MBC). The minimal fungicidal concentration (MFC) value was defined as the lowest plant extract concentration that shows no visible fungal growth. Tubes that were just inoculated with microorganisms and tubes that were only inoculated with media served as controls. All the experiments were carried out in triplicate, and the results were recorded in a table.

### 2.8. Statistical Analysis

All experiments were performed in triplicates. Data were analyzed by GraphPad Prism version 9.0.0 and the results are provided as mean ± SEM. One-way analysis of variance (ANOVA) and post hoc ANOVA using Tukey's HSD test with a 95% confidence level was used to compare the differences in the mean zone of inhibitions among and between the groups, respectively. Differences among groups were statistically significant at *p* < 0.05.

### 2.9. Ethical Approval

The study was performed after obtaining institutional ethical approval from the Faculty of Veterinary Medicine Biosafety; Animal Use and Ethics Committee (BAUEC) of the University of Nairobi ethical approval, reference number FVM BAUEC/2021/290; and a research permit from the National Commission for Science, Technology, and Innovation (NACOSTI), license number NACOSTI/P/21/11253.

## 3. Results

### 3.1. Phytochemical Composition

Aqueous extracts had the highest yield value (7%), followed by methanol with 2.3% and the mixture of dichloromethane : methanol (1 : 1) recorded the least (1.89% yield value). The three extracts and the powder of *D. abyssinica* rhizome possessed secondary metabolites (Table 1). Except for the aqueous extract, saponins were detected in the powder and all other extracts. Alkaloids, glycosides, phenolics, coumarins, tannins, flavonoids, steroids, and terpenoids were present in the powder and all other extracts (aqueous dichloromethane : methanol (1 : 1) and methanol) from *D. abyssinica* rhizome. Mayer's test for alkaloids was negative while Dragendorff's test was positive for all the samples.

### 3.2. Effects of Single-Dose Toxicity

The study of the toxic effects of *D. abyssinica* aqueous, methanol, and dichloromethane : methanol extracts after oral single-dose administration revealed no signs of unwellness (Table 2). In addition, there wasn't any rat mortality that was observed at a single dose of 2,000 mg/kg body weight (bw). Therefore, the extracts were classified as nontoxic according to the OECD 425 guidelines. The LD_50_ of the extracts was found to be more than 2000 mg/kg. To the best of our knowledge, this was the first time the toxicity profile of *D. abyssinica* is being reported.

### 3.3. Antimicrobial Activity

The crude extracts of *D. abyssinica* rhizome were tested for antimicrobial efficacy against four pathogenic microorganisms utilizing disk diffusion and microdilution techniques. The study revealed that the extracts had antifungal activity and did not demonstrate antibacterial activity against the studied microorganisms. Tables 3 and 4 summarize that the aqueous, methanol, and dichloromethane : methanol (1 : 1) extracts revealed antifungal activity against *C. albicans*. The highest antifungal activity was demonstrated by aqueous extract which had a zone of inhibition of 16.33 ± 0.82 (Table 3) at a concentration of 500 mg/ml and a MIC of 31.25 mg/ml (Table 4). All three extracts (aqueous, methanol, and dichloromethane : methanol (1 : 1) were inactive against the bacterial microorganisms of *E. coli*, *N. gonorrhea*, and *S. aureus.*

## 4. Discussion

Medicinal plants are endowed with secondary metabolites. These plants continue to provide solutions to human and livestock ailments in traditional medicine systems for primary health care. In the current study, chemical compounds known to have antimicrobial activities were detected in the rhizome extracts of *D. abyssinica*. Phenols, tannins, flavonoids, terpenoids, saponins, alkaloids, and coumarins which were positively indicated in the aqueous, methanol, and dichloromethane : methanol (1 : 1) extracts are known to have antimicrobial activities and may be explored to develop botanicals that can be used to combat antimicrobial resistance [48]. To the best of our knowledge, this is the first published report on the qualitative phytochemical composition of the powder and extracts from *D abyssinica* rhizome. Also, toxic effects and antimicrobial activity studies have not been reported in the literature. However, reports exist for several plants from the *Digitaria* genus that possesses active phytochemicals with antimicrobial activities and other varied biological activities including analgesic, antiviral, anti-inflammatory, antitumor, anthelmintic action, and effects on the central nervous system [49, 50].

All female Wistar rats in this study, survived after administration of a single dose of 2000 mg/kg of the aqueous, methanol, and dichloromethane : methanol (1 : 1) extracts of *D. abyssinica* to the Wistar rats orally. Therefore, the dose of the *D. abyssinica* rhizome extracts that can kill half of the tested animals was more than 2000 mg/kg. According to the Organization for Economic Cooperation and Development (OECD) test no 425, the findings in this study indicate that the extracts are not toxic to the female Wistar rats as in many other acute toxicity studies of herbal medicines [45, 51, 52]. The current research indicates that *D. abyssinica* rhizome extracts have antifungal activity against *C. albicans* and that the strength of the activity was directly proportional to the concentration of the extract loaded to the disk. The size of the zones of inhibition of the aqueous extract was more than 15 mm at concentrations 125 mg/ml, 250 mg/ml, and 500 mg/ml (Table 3) which is interpreted as potential antifungal activity [53, 54]. There was no statistical difference in activity at concentrations more than 125 mg/ml at *p* < 0.05 (Table 3). Though there was noted low activity at 15.625 mg/ml, 31.25 mg/ml, and 62.50 mg/ml of zones of inhibition between 6.5 mm and 9.67 mm, there were statistical differences in activity at *p* < 0.05.

The methanol extract also had high antifungal activity (15.17 ± 0.75 mm) at a concentration of 500 mg/ml and is statistically different from the activity of 11.67 ± 0.52 mm exerted by the extract at a concentration of 250 mg/ml at *p* < 0.05. Subsequently, zones of inhibition of 11.50 ± 0.55, 8.83 ± 0.75, 8.33 ± 0.52, and 6.83 ± 0.41 mm at concentrations of 125, 62.50, 31.25, and 15.625 mg/ml, respectively. There were notable statistical differences between the antifungal activities of the methanol extract at concentrations between 15.625 and 31.25 mg/ml (*p* < 0.05) and between 62.50 and 125 mg/ml. However, there were no statistical differences in activities between concentrations 31.25 and 62.50 mg/ml and between concentrations 125 and 250 mg/ml at *p* < 0.05. The zones of inhibition that revealed the activity of the mixture of dichloromethane : methanol *D. abyssinica* rhizome extract similarly reflected proportionality to extract concentrations (Table 3). The aqueous, methanol, and dichloromethane : methanol (1 : 1) extracts of *D. abyssinica* did not show any zone of inhibition against the bacterial strains, *E. coli*, *N. gonorrhoeae*, and *S. aureus*, in this study.

## 5. Conclusion and Recommendation

Based on the current study, it was concluded that the aqueous, methanol, and mixture of dichloromethane : methanol (1 : 1) extracts of *D. abyssinica* rhizome possess phytochemicals compounds with anticandidal activity. The extracts can be classified as nontoxic and therefore safe for female Wistar rats when administered orally at a single dose. It was further deduced that the extracts had low antifungal effects. Nevertheless, herbalists and traditional medicine practitioners have used the plant material in wide cultural setups. Given that herbalist frequently uses aqueous extracts in their practice, these study findings provide scientific evidence to validate the use of the extracts in the management of candidiasis. Bioassay-guided fractionation was recommended to isolate anticandidal compounds from *D. abyssinica* rhizome extracts. In addition, quantitative phytochemical studies and subchronic and chronic toxicity assays were recommended.

## Figures and Tables

**Table 1:** Phytochemical composition of *D*. *abyssinica* rhizome powder and extracts.

Phytochemicals	Method/reagent	Powder	Aqueous	Methanol	Dichloromethane : methanol (1 : 1)
Saponins	Foam test	+	−	+	+
Phenolics	Ferric chloride	+	+	+	+
Alkaloids	Mayer's Drangendorff's	− +	− +	− +	− +
Cardiac glycosides	Keller–Killiani Keddie	+ +	+ +	+ +	+ +
Tannins	Ferric chloride	+	+	+	+
Coumarins	Ferric chloride	+	+	+	+
Flavonoids	NaOH	+	+	+	+
Steroids	Liebermann–Burchard	+	+	+	+
Terpenoids	Salkowski	+	+	−+	+

Key: (+)—present; (−)—absent.

**Table 2:** Showing effects of *D. abyssinica* extracts on female Wistar rats wellness parameters and LD_50_ at 2000 mg/kg oral single-dose.

Wellness parameter	Observation after											
	30 minutes		4 hours		24 hours		48 hours		7 days		14 days	
	EGR	CGR	EGR	CGR	EGR	CGR	EGR	CGR	EGR	CGR	EGR	CGR
Skin and fur appearance	Normal	Normal	Normal	Normal	Normal	Normal	Normal	Normal	Normal	Normal	Normal	Normal
Fecal matter consistency	Normal	Normal	Normal	Normal	Normal	Normal	Normal	Normal	Normal	Normal	Normal	Normal
Urination and urine appearance	Normal	Normal	Normal	Normal	Normal	Normal	Normal	Normal	Normal	Normal	Normal	Normal
Mucous membrane appearance	Normal	Normal	Normal	Normal	Normal	Normal	Normal	Normal	Normal	Normal	Normal	Normal
Itching	Absent	Absent	Absent	Absent	Absent	Absent	Absent	Absent	Absent	Absent	Absent	Absent
Salivation	Normal	Normal	Normal	Normal	Normal	Normal	Normal	Normal	Normal	Normal	Normal	Normal
Convulsions and tremors	Absent	Absent	Absent	Absent	Absent	Absent	Absent	Absent	Absent	Absent	Absent	Absent
Breathing	Normal	Normal	Normal	Normal	Normal	Normal	Normal	Normal	Normal	Normal	Normal	Normal
Coma	Absent	Absent	Absent	Absent	Absent	Absent	Absent	Absent	Absent	Absent	Absent	Absent
Somatomotor activity	Normal	Normal	Normal	Normal	Normal	Normal	Normal	Normal	Normal	Normal	Normal	Normal
Aggression	Absent	Absent	Absent	Absent	Absent	Absent	Absent	Absent	Absent	Absent	Absent	Absent
Grooming	Normal	Normal	Normal	Normal	Normal	Normal	Normal	Normal	Normal	Normal	Normal	Normal
Eyes	Normal	Normal	Normal	Normal	Normal	Normal	Normal	Normal	Normal	Normal	Normal	Normal
Mortality/death	None	None	None	None	None	None	None	None	None	None	None	None

Key: EGR—experimental group of female Wistar rats (administered with the studied plant extracts); CGR—control group of female Wistar rats (administered with physiological saline only).

**Table 3:** Mean diameter zone of inhibition of *Digitaria abyssinica* rhizome extracts and controls using disk diffusion method.

Zone of inhibition (mm)						
Pathogen	Concentration (mg/mL)	Aqueous extract	Methanol extract	Dichloromethane : methanol extract	Negative control	Positive control
*Candida albicans*	15.625	6.50^a^ ± 0.55	6.83^a^ ± 0.41	0.00^a^ ± 0.00	0.00^a^ ± 0.00	Clotrimazole: 27.00^f^ ± 0.89
	31.25	7.83^b^ ± 0.75	8.33^b^ ± 0.52	6.83^b^ ± 0.41		Econazole: 26.67^f^ ± 0.52
	62.50	9.67^c^ ± 0.52	8.83^b^ ± 0.75	8.17^c^ ± 0.41		Ketoconazole: 34.17^g^ ± 0.75
	125.00	15.50^d^ ± 0.55	11.67^c^ ± 0.52	9.50^d^ ± 0.55		Miconazole: 24.83^e^ ± 0.75
	250.00	15.67^d^ ± 0.52	11.50^c^ ± 0.55	9.50^d^ ± 0.55		Nystatin: 7.17^ab^ ± 0.41
	500.00	16.33^d^ ± 0.82	15.17^d^ ± 0.75	10.50^e^ ± 0.55		

*Escherichia coli*	15.625	0.00^a^ ± 0.00	0.00^a^ ± 0.00	0.00^a^ ± 0.00	0.00^a^ ± 0.00	Azithromycin: 6.00^b^ ± 0.00
	31.25	0.00^a^ ± 0.00	0.00^a^ ± 0.00	0.00^a^ ± 0.00		Ceftriaxone: 29.00^e^ ± 0.89
	62.50	0.00^a^ ± 0.00	0.00^a^ ± 0.00	0.00^a^ ± 0.00		Cephalexin: 12.33^c^ ± 0.52
	125.00	0.00^a^ ± 0.00	0.00^a^ ± 0.00	0.00^a^ ± 0.00		Ciprofloxacin: 30.33^f^ ± 0.82
	250.00	0.00^a^ ± 0.00	0.00^a^ ± 0.00	0.00^a^ ± 0.00		Tetracycline: 18.67^d^ ± 0.52
	500.00	0.00^a^ ± 0.00	0.00^a^ ± 0.00	0.00^a^ ± 0.00		

*Neisseria gonorrhoeae*	15.625	0.00^a^ ± 0.00	0.00^a^ ± 0.00	0.00^a^ ± 0.00	0.00^a^ ± 0.00	Azithromycin: 21.00^b^ ± 0.71
	31.25	0.00^a^ ± 0.00	0.00^a^ ± 0.00	0.00^a^ ± 0.00		Ceftriaxone: 28.50^c^ ± 4.23
	62.50	0.00^a^ ± 0.00	0.00^a^ ± 0.00	0.00^a^ ± 0.00		
	125.00	0.00^a^ ± 0.00	0.00^a^ ± 0.00	0.00^a^ ± 0.00		
	250.00	0.00^a^ ± 0.00	0.00^a^ ± 0.00	0.00^a^ ± 0.00		
	500.00	0.00^a^ ± 0.00	0.00^a^ ± 0.00	0.00^a^ ± 0.00		

*Staphylococcus aureus*	15.625	0.00^a^ ± 0.00	0.00^a^ ± 0.00	0.00^a^ ± 0.00	0.00^a^ ± 0.00	Cephalexin: 15.50^c^±0.55
	31.25	0.00^a^ ± 0.00	0.00^a^ ± 0.00	0.00^a^ ± 0.00		Ciprofloxacin:22.17^d^ ± 0.75
	62.50	0.00^a^ ± 0.00	0.00^a^ ± 0.00	0.00^a^ ± 0.00		Tetracycline:11.33^b^ ± 0.52
	125.00	0.00^a^ ± 0.00	0.00^a^ ± 0.00	0.00^a^ ± 0.00		
	500.00	0.00^a^ ± 0.00	0.00^a^ ± 0.00	0.00^a^ ± 0.00		

Values are expressed as mean ± SD of three separate determinations. Means with different superscripts along the column are significantly different at *p* *<* 0.05.

**Table 4:** Average MIC and MBC or MFC (mg/ml) of *D. abyssinica* extracts against selected uropathogenic microorganisms.

Test organism	Aqueous extract		Methanol extract		Dichloromethane : methanol extract	
	MIC	MBC or MFC	MIC	MBC or MFC	MIC	MBC or MFC
*Candida. albicans*	31.25	62.5^MBC^	31.25	62.5^MBC^	62.5	125^MBC^
*Escherichia coli*	>250	>250^MBC^	>250	>250^MBC^	>250	>250^MBC^
*Neisseria gonorrhoeae*	>250	>250^MBC^	>250	>250^MBC^	>250	>250^MBC^
*Staphylococcus aureus*	>250	>250^MFC^	>250	>250^MFC^	>250	>250^MFC^

Key: MIC—minimum inhibitory concentration; MBC—minimum bactericidal concentration; MFC—minimum fungicidal concentration.

## Data Availability

All data underlying the results are available as part of the article and no additional source data are required.
